# Taxonomic studies of *Glochidion* (Phyllanthaceae) from the Indo-China Peninsula (II): The identities of *G.anamiticum* and *G.annamense*

**DOI:** 10.3897/phytokeys.114.30725

**Published:** 2018-12-13

**Authors:** Gang Yao, Zhuqiu Song, Youheng Wu, Nguyen Thi Kim Thanh, Yuling Li, Shi Shi, Shixiao Luo

**Affiliations:** 1 South China Limestone Plants Research Center, College of Forestry and Landscape Architecture, South China Agricultural University, Guangzhou 510642, China South China Agricultural University Guangzhou China; 2 Key Laboratory of Plant Resources Conservation and Sustainable Utilization, South China Botanical Garden, Chinese Academy of Sciences, Guangzhou 516650, China South China Botanical Garden, Chinese Academy of Sciences Guangzhou China; 3 University of Chinese Academy of Sciences, Beijing 1000049, China University of Chinese Academy of Sciences Beijing China; 4 Department of Botany, Faculty of Biology, HNU Herbarium, VNU University of Science, Vietnam VNU University of Science Hanoi Vietnam

**Keywords:** *
Glochidion
*, Phyllanthaceae, Taxonomy, Vietnam

## Abstract

The names *Glochidionanamiticum* (Kuntze) Kuntze ex Merrill and *G.annamense* Beille were previously reduced to synonyms of *G.eriocarpum* Champ. ex Benth. However, literature examination and morphological comparison suggest that *G.annamense* is conspecific with *G.anamiticum* and the species can be readily distinguished from *G.eriocarpum* by its styles connate into a long cylindric column and up to 3 mm long, ovaries usually 3-locular, stigma usually 3-lobed, capsules pubescent and usually 6-grooved, persistent styles 3‒4 mm long. Thus, the specific status of *G.anamiticum* is here reinstated and G. *annamense* is treated as its synonym.

## Introduction

*Glochidion* J. R. Forster & G. Forster is a large genus within the tribe Phyllantheae Dumortier, family Phyllanthaceae Martinov (Webster 2014, APG 2016). It consists of over 300 species mainly distributed in the Indo-Pacific region, east to southeast Polynesia and south into Australia (Webster 2014, Yao and Zhang 2016). The genus can be distinguished from all the other genera in the tribe by its usually unlobed styles, apiculate anthers and usually fleshy seed coat (Webster 2014).

Molecular phylogenetic studies revealed that *Glochidion*, together with *Breynia* J. R. Forster & G. Forster, *Phyllanthodendron* Hemsl. and *Sauropus* Blume, were all deeply embedded within *Phyllanthus* L. s. str. (Hoffmann et al. 2006, Kathriarachchi et al. 2006). So, some authors suggested the merger of these genera with *Phyllanthus* and accepted the broad concept of *Phyllanthus* s. l. (including *Breynia*, *Glochidion*, *Phyllanthodendron* and *Sauropus*) as a super-genus that includes over 1,200 species (Hoffmann et al. 2006). However, Pruesapan et al. (2012) suggested that the reinstatement of *Breynia*, *Glochidion*, *Phyllanthodendron* and *Sauropus*, as well as the disintegration of the paraphyletic *Phyllanthus* s. str. (over 800 species) into smaller genera, seems to be more reasonable, because these groups can be distinguished easily from each other in morphology and *Phyllanthus* s. str. is actually a large and morphologically heterogeneous genus with wide distribution areas. The latter suggestion is further supported by morphological (van Welzen et al. 2014), palynological (Yao and Zhang 2016) and wood anatomical (Jangid and Gupta 2016) studies. Thus, the acceptance of the generic status of *Glochidion* is widely adopted in most recent literature (e.g. van Welzen et al. 2014, Webster 2014, Duocet Group 2016 onwards, Kato and Kawakita 2017).

The species *Glochidionanamiticum* (Kuntze) Kuntze ex Merrill was originally described by Kuntze (1891) in the genus *Diasperus* L. ex Kuntze based on one collection (*O. Kuntze 3798*) from Vietnam and *Glochidionannamense* Beill was described by Beille (1927) based on five collections (*F. Evrard 233*, *C. Gaudichaud 161*, *M. Krempf 1567*, *E. Poilane 7838* and *E. Poilane 10022*) from Vietnam, too. The latter was soon reduced to the synonymy of *G.anamiticum* (Merrill 1936). In the taxonomic study of the Thai Euphorbiaceae, Airy Shaw (1972) provisionally placed both names under *G.eriocarpum* Champ. ex Benth., expressing uncertainty by question marks and this questionable treatment was directly followed by van Welzen (2007) in Flora of Thailand. Subsequent taxonomists accepted Airy Shaw’s proposal to treat G.anamiticum and G. *annamense* as synonyms of *G.eriocarpum* (Govaerts et al. 2000, Li and Gilbert 2008, The Plant List 2013 onwards, Govaerts et al. 2018). However, *G.annamense* was recognised as a separate species by Nguyen (2007) when treating the Vietnamese Euphorbiaceae, but *G.anamiticum* was not considered. In our comprehensive taxonomic studies of *Glochidion*, we found that *G.anamiticum* and *G.annamense* represent the same species, but this species is clearly different from *G.eriocarpum* in morphology. We therefore propose to reinstate the specific status of *G.anamiticum* and treat *G.annamense* as a synonym of *G.anamiticum*.

## Material and methods

Specimens of *Glochidion* deposited in the herbaria HN, IBSC, K, KUN, NY, P and PE were studied carefully in the present study and field investigations of *G.anamiticum* and *G.eriocarpum* were carried out in recent years. Morphological characters of relevant species were also photographed and some of them were measured. Herbarium abbreviations cited here follow the Index Herbariorum (Thiers 2018 onwards).

## Results and discussions

**Figure 1.**
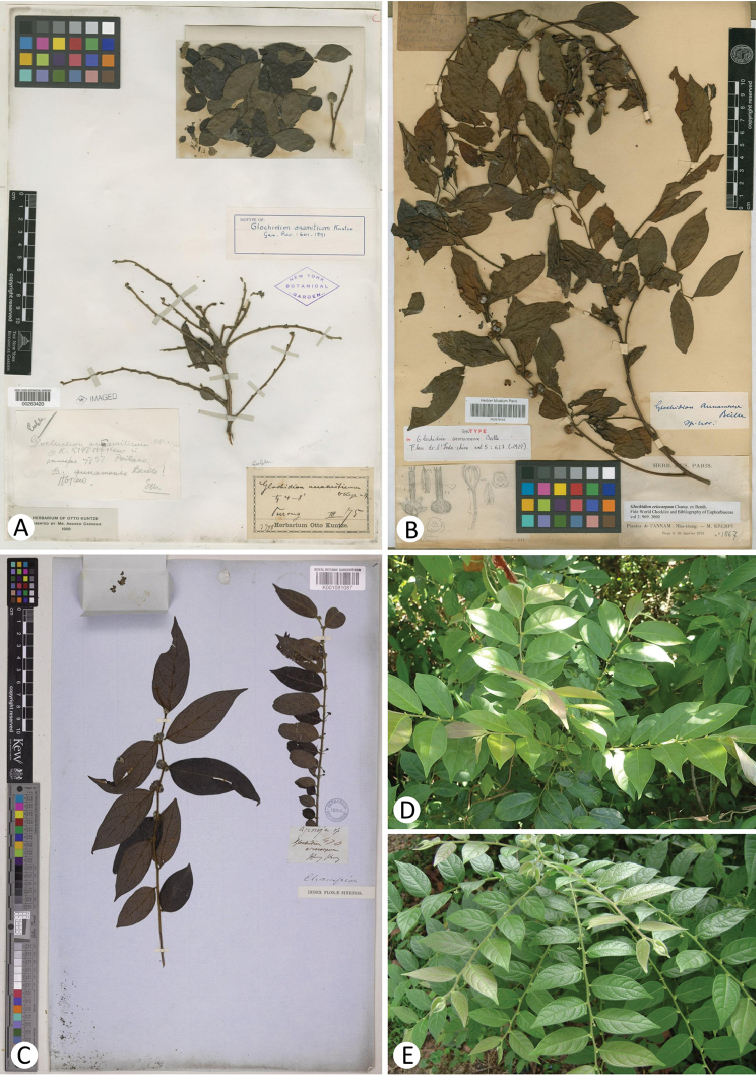
Type (**A–C**) and field images (**D–E**) of *Glochidion* species. **A** Lectotype of *G.anamiticum* (Kuntze) Kuntze ex Merrill (*O. Kuntze 3798*, NY-00263420) **B** Syntype of *G.annamense* Beille (*M. Krempf 1567*, P-00379152) **C** Type of *G.eriocarpum* Champ. ex Benth. (*J.G. Champion 470*, K-001081087) **D***G.anamiticum*; E, *G.eriocarpum*.

**Figure 2.**
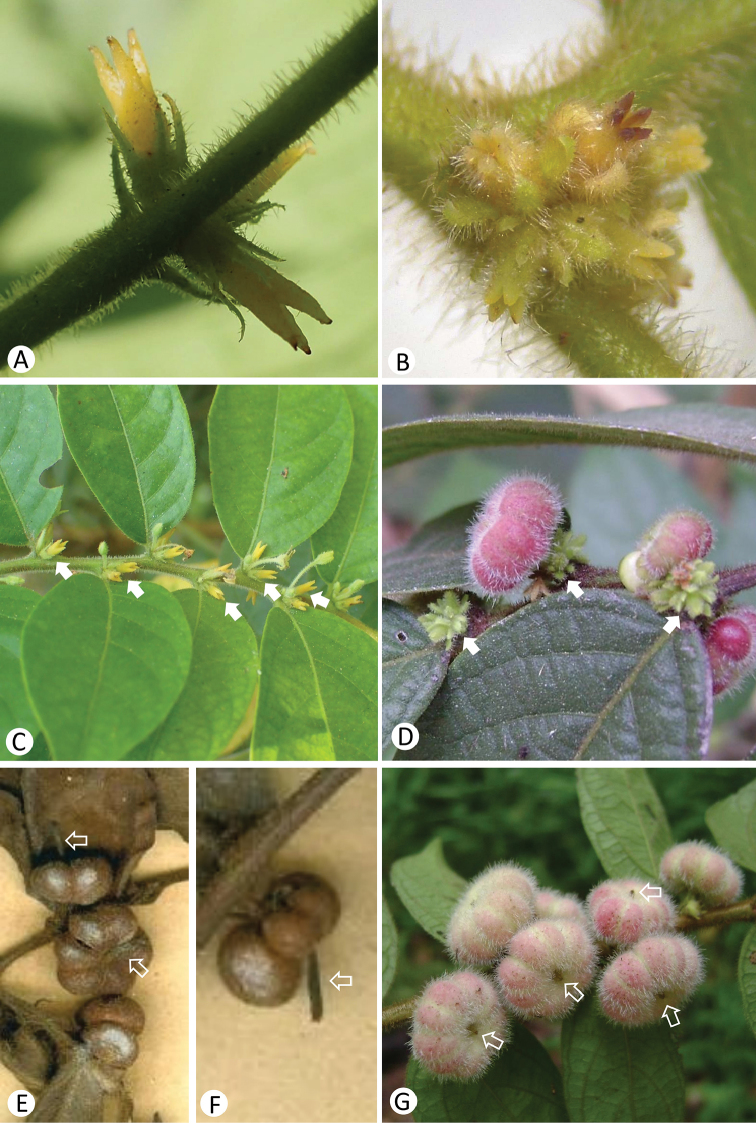
Morphological comparisons between *Glochidionanamiticum* (Kuntze) Kuntze ex Merrill (**A, C, E, F**) and *G.eriocarpum* Champ. ex Benth. (**B, D, G**). **A–D** Female flower **D–G** Fruit. The female flowers and persistent styles in the fruits are shown by the solid arrowhead and hollow arrowhead, respectively.

*Glochidionanamiticum* (Figures 1A and D) and *G.annamense* (Figure 1B) represent the same species, which is similar to *G.eriocarpum* (Figures 1C and E) in habit to some extent. However, the Vietnamese endemic species *G.anamiticum* is morphologically distinct and can be readily distinguished from *G.eriocarpum* (Table 1) by its stipules triangular to narrowly triangular and ca. 1‒1.5 mm long (Figure 2A) (vs. narrowly triangular to linear, 1‒4 mm long), sepals of female flowers ovate-triangular to narrowly triangular and pubescent outside (Figures 2A and C) (vs. oblong and villous outside; Figures 2B and D), styles connate into a long cylindrical column up to 3 mm long (Figures 2A and C) (vs. a cylindrical column 1‒1.5 mm long; Figure 2B), ovaries usually 3-locular (vs. usually 4‒5-locular), stigma usually 3-lobed (Figure 2A) (vs. usually 4‒5-lobed; Figure 2B), capsules pubescent and usually 6-grooved (Figures 2E and F) (vs. villous and usually 8‒10-grooved; Figure 2G), persistent style cylindrical column 3‒4 mm long (Figures 2E and F) (vs. shortly column-shaped to subconical, less than 1 mm long; Figure 2G). Thus, our results suggest that it may be best to treat *G.anamiticum* and *G.eriocarpum* as separate species. The following taxonomic treatment is therefore necessary.

**Table 1.** Morphological comparisons between *Glochidionanamiticum* (Kuntze) Kuntze ex Merrill and *G.eriocarpum* Champ. ex Benth.

Traits/Species	* Glochidion anamiticum *	* G. eriocarpum *
Stipule	Triangular to narrowly triangular; ca. 1‒1.5 mm long;	Narrowly triangular to linear; 1‒4 mm long;
Sepals of female flower	Ovate-triangular to narrowly triangular; pubescent outside;	Oblong; villous outside;
Style	Cylindrical column, long exceeding the sepals, 2‒3 mm long;	Cylindrical column, slightly exceeding the sepals, 1‒1.5 mm long;
Stigma	Deeply 3(4)-lobed;	Deeply 4‒5-lobed;
Ovary	Pubescent; 3(4)-locular;	Villous; 4‒5-locular;
Capsule	Pubescent; 7‒9 mm in diameter; deeply 6(8)-grooved;	Villous; 8‒10 mm in diameter; deeply 8‒10-grooved;
Persistent style	Cylindrical column; up to 4 mm long;	Shortly column-shaped to subconical; less than 1 mm long.

## Taxonomic treatment

### Glochidion anamiticum

Taxon classificationPlantaeMalpighialesPhyllanthaceae

(Kuntze) Kuntze ex Merrill, Brittonia 2: 195. 1936

Figures 1A, B, D , 2A, C, E, F

#### Basionym.

*Diasperusanamiticus* Kuntze, Revis. Gen. Pl. 2: 601. 1891.

#### Type.

Vietnam. Annam, Turong, March 1875, *O. Kuntze 3798* (lectotype designated by Merrill (1936: 195): NY-00263420, photo!; isolectotype: K-001081193, photo!).

*Glochidionannamense*Beille (1927, p. 627). Type: Vietnam. Annam, Lien-chien, 25 August 1923, *E. Poilane 7838* (lectotype designated by Nguyen (2007): HM; isolectotypes: K-001081192, P-00379159, P-00379160, photos!). Remaining syntypes: Vietnam. Annam, Trai-ca, province, Phanrang, 9 March 1924, *E. Poilane 10022* (P-00379161, photo!); Tourane, January 1837, *C. Gaudichaud 161* (P-00379151, photo!); Nha-trang, *M. Krempf 1567* (P-00379152, photo!); Dalat réserve du Calmy, 24 October 1920, *F. Evrard 233* (P-00379150, photo!).

Shrubs, 1‒3 m tall, monoecious; branchlets usually pubescent; leaf blade membranous, 6‒8 × 2.5‒3 cm, lanceolate, oblong to ovate, sometimes asymmetrical, base acute to round, apex acuminate, adaxially dark brown or blackish, pubescent on the median rib, abaxially light brown, pubescent; lateral veins 6‒8 pairs, elevated abaxially; petiole ca. 2‒3 mm long, pubescent; stipules triangular to narrowly triangular, 1‒1.5 mm long. Male flowers: pedicels ca. 10 mm long, pubescent; sepals 6, oblong to ovate, hairy outside, glabrous inside; stamens 3, connate, surmounted by a conical prolongation. Female flowers: in axillary clusters, subsessile; sepals 6, ca. 1.2 mm long, ovate-triangular or narrowly triangular, hairy outside, glabrous inside; ovary depressed globose, 3 (rarely 4)-locular, pubescent; style cylindrical column, long exceeding the sepals, 2‒3 mm long, hairy in the lower quarter, glabrous in the upper part, 3 (rarely 4)-lobed at the top. Capsule depressed globose, 6 (rarely 8)-grooved, 7‒9 mm in diameter, ca. 5 mm high, very briefly hairy; persistent style a long cylindrical column up 3‒4 mm long; fruit pedicels stout, 3‒4 mm long, pubescent.

#### Distribution and habitat.

*Glochidionanamiticum* is endemic to Vietnam, mostly from Da Nang to Ninh Thuan (Nguyen 2007).

#### Notes.

In the monograph *Revisio Generum Plantarum*, Kuntze (1891) transferred hundreds of *Glochidion* and *Phyllanthus* species into the genus *Diasperus* L. ex Kuntze and he also described many new species, including *Diasperusanamiticus* Kuntze which was based on one of Kuntze’s collections (*O. Kuntze 3798*) from Vietnam. At the same time, however, a specific name (*Glochidionanamiticum*), under the generic name *Glochidion*, was also suggested for *D.anamiticus*, but it was listed as a synonym of the new species. According to Art. 34.1 of the ICN (McNeill et al. 2012), the name *G.anamiticum* Kuntze is invalid because it was not accepted by the author and just cited as a synonym of *Diasperusanamiticus*. On the other hand, many of the new species described by Kuntze (1891) were overlooked for a long time, until they were re-evaluated by Merrill (1936) and then the species *Glochidionanamiticum* was accepted formally as a member of *Glochidion* for the first time. So, the author’s attribution of the species should be *Glochidionanamiticum* (Kuntze) Kuntze ex Merrill.

In his commentary on Loureiro’s (1790) new species described from southern Vietnam, Merrill (1935) transferred the species *Nymphanthuspilosus* Lour. into *Glochidion* and reduced *Glochidionannamense* to a synonym of his new combination *Glochidionpilosum* (Lour.) Merr., although the type of *G.pilosum* was not examined in his study. Merrill’s (1935) taxonomic treatment of *Glochidionannamense* was followed by Ho (2003) in An Illustrated Flora of Vietnam. However, as noted by Loureiro (1790) in the protologue of *Nymphanthuspilosus*, the species was characterised by pinnate leaves and bacciformis fruits, which are much different from those of the genus *Glochidion* (characterised by alternate and distichous leaves, fruits capsule), but consistent with those of *Emblica* Gaertn. or Phyllanthussect.Emblica, thus the species also had been transferred into these two genera and named as *Emblicapilosa* (Lour.) Spreng. or *Phyllanthuspilosus* (Lour.) Müll. Arg., respectively. In the following year, however, Merrill (1936) treated the name *Glochidionannamense* as a synonym of *G.anamiticum*, based on the study of type specimens, but the name *G.pilosum* was not considered.

Morphological comparisons between *Glochidionanamiticum* and *G.eriocarpum* can be seen in Table 1 and Figures 1, 2. The most noticeable traits of *G.anamiticum* are its long style (up to 3 mm long) in the female flowers and long persistent style (up to 4 mm long) in the fruits.

#### Additional specimens examined.

***Glochidionanamiticum***: VIETNAM. Detailed locality unknown, 7 April 1978, *Nhan 294* (HN); Annam, Hué, Flower pale yellow; Near river, 11 March 1927, *R.W. Squires 178* (P-00379167); Arbre Broyé (Tuyen Duc), altitude 1500 m, 29 March 1953, *M. Schmid s.n.* (P-00509785); Dalat, ravin au S. du Langbian Palace, 17 May 1924, *F. Evrard 910* (P-00379154); Dalat, lac des Soupirs, altitude 1500 m, 12 January 1964, *M. Schmid s.n.* (P-00509783); Dalat, ravin derrière la gendarmerie, 2 October 1924, *F. Evrard 1293* (P-00379155); Dalat, ravin au Sud du Langbian Palace, 14 November 1924, F. Evrard 1788 (P-00379156); Région de Dalat, September 1952, *M. Schmid 1288* (P-00509784); Cochinchine, prov. de Bienhoa, 11 October 1931, *E. Poilane 19574* (P-00379164); Env. de Huê, prov. Thua Thiên-Huê, 23 December 1943, *J.E. Vidal 917a* (P-00476551). Hue, Bach Ma National Park, 3 August 2018, *Y.H. Wu 17126* (IBSC). ***Glochidioneriocarpum***: CHINA. Guangdong Province, Jiangmen, 21 October 2013, *L.X. Zhou 11542* (KUN); Guangdong Province, Yangchun, Ehuangzhang Nature Reserve, 14 November 2008, *G. Yao 018* (IBSC); Guangdong Province, Zhaoqing, Dinghushan Nature Reserve, 17 December 2008, *G. Yao 058* (IBSC); Guangxi Province, Rongshui, 24 October 2008, *G. Yao 08-008* (IBSC); Hainan Province, Xinglong, 6 March 2009, *G. Yao 101* (IBSC); Hongkong, J.G. Champion 470 (K); Yunan Province, Puer, 1 April 2003, *Y. Chen s.n.* (KUN); Yunan Province, Xishuangbanna, 29 July 1977, *G.D. Tao 15679* (KUN); VIETNAM. Annam, Environs du poste forestier de Bang Trê Lat sur sông Con, prov. de Vinh, 24 July 1929, *E. Poilane 16419* (P-00379163); Hanh Hoa Province, Thach Thanh District, Cuc Phuong NP, Thanh Yenh Commune, Sanh Village, altitude 150 m, 27 February 2001, *N.M. Cuong 1320* (P-00520940); Tonkin, Binh Lei, 43 km à l’ouest de Chapa, altitude 1000–1200 m, 12 August 1926, *E. Poilane 12899* (P-00379162); Tonkin, Taai Wong Mo Shan and vicinity, Ton fa market, Ha-coi, September 1939, *W.T. Tsang 29570* (P-00379168).

## Supplementary Material

XML Treatment for Glochidion anamiticum
